# Bacterial isolates and their antimicrobial susceptibility pattern among patients with external ocular infections at Borumeda hospital, Northeast Ethiopia

**DOI:** 10.1186/s12886-015-0078-z

**Published:** 2015-08-14

**Authors:** Birtukan Shiferaw, Baye Gelaw, Abate Assefa, Yared Assefa, Zelalem Addis

**Affiliations:** School of Biomedical and Laboratory sciences, College of Medicine and Health Sciences (CMHS), University of Gondar (UOG), P.O. box 196, Gondar, Ethiopia; Department of Medical Microbiology, School of Biomedical and Laboratory sciences, CMHS, UOG, P.O. box 196, Gondar, Ethiopia; Department of Ophthalmology, School of Medicine, CMHS, UOG, P.O. box 196, Gondar, Ethiopia

**Keywords:** Bacterial isolate, External ocular infections, Drug susceptibility pattern

## Abstract

**Background:**

Bacteria are major cause of ocular infections and possible loss of vision. The emergence of antimicrobial resistant bacteria increases the risk of treatment failure with potentially serious consequences. The aim of this study was to determine the prevalence of bacterial isolates and their antimicrobial susceptibility pattern among patients with external ocular infections.

**Methods:**

A cross sectional study was conducted among 160 patients with external ocular infections at Borumeda hospital, Northeast Ethiopia. Socio-demographic and clinical data were collected using structured questionnaire. External ocular specimens were collected using sterile swabs and inoculated on MacConkey agar, Chocolate agar and Blood agar culture Medias. Presumptive isolates were further identified by a series of biochemical tests. The antimicrobial susceptibility patterns of the isolates were determined by disk diffusion method.

**Result:**

The overall prevalence of bacterial pathogens among external ocular samples was 59.4 %. The majority of the isolates (93.7 %; 89/95) were Gram positive and the other 6.3 % (6/95) Gram negative bacteria. The proportion of coagulase negative *Staphylococci* among the Gram positive bacterial isolates was 53.7 % (*n* = 51/95). All Gram positive isolates were susceptible for vancomycin but 67.4 % (*n* = 60/95) of them were resistant against amoxicillin. Moreover, drug resistance to tetracycline, norfloxacylin, ceftriaxone and ciprofloxacin were observed among Gram negative bacteria isolates.

**Conclusion:**

The prevalence of bacterial pathogens among external ocular samples was high and the predominant isolate was coagulase negative *Staphylococci*. Exceptionally high amoxicillin resistance was observed among Gram positive bacterial isolates that may dictate to conduct drug susceptibility test routinely.

## Background

Pathogenic micro-organisms cause ocular disease and the most frequently affected parts of the eye are the conjunctiva, lid and cornea [1]. Bacteria are major causative agents that frequently cause infections in eye and possible loss of vision [2]. Frequently reported clinical manifestations include conjunctivitis, scleritis, keratitis, blepharitis, canaliculitis and dacryocystitis [3]. Conjunctivitis is the most common cause of “red eye” [4] and corneal ulceration is a major cause of mono-ocular blindness in developing countries [5]. The three most common causes of conjunctivitis are infection (infective conjunctivitis), allergic reactions and irritation (loose eyelash). Infective conjunctivitis is most commonly caused by bacteria and viruses. Viral conjunctivitis causes a watery discharge while the discharge from bacterial conjunctivitis contains pus [6]. Infective keratitis is a major cause of vision loss and blindness second to cataract [7, 8]. Blepharitis is an inflammation of the eyelid margins which can result in patient discomfort and decline in visual function [9] while endophthalmitis may cause vision-threatening ocular complications following intraocular surgeries and during an open-globe injuries. Dacryocystitis is an inflammation of the lacrimal sac and duct [10, 11].

The morbidity due to ocular infections can vary from self limiting, trivial infection to sight threatening and blindness [12]. A variety of factors determine clinical outcome in microbial caused eye infection and the epidemiological patterns vary from one country to the other and in different geographical areas in the same country [13]. In Ethiopia the prevalence of blindness was reported about 1.6 % and it was estimated that 87.4 % of the cases were due to avoidable causes [14].

The management of bacterial eye infections may involve treatment with broad spectrum antibiotics. The indiscriminate use of antibiotics led to the development of resistance to many commonly used antimicrobial medications. The emergence of bacterial resistance towards topical antimicrobial agents may increases the risk of treatment failure with potentially serious consequences [3]. Therefore, up to date information is essential for appropriate antimicrobial therapy and management of ocular infections [15]. We believe that the spectrum of bacterial agents responsible for external ocular infections and their drug susceptibility pattern in the study area as well as in Ethiopia are not well reported or there is a scarcity of published data. Thus, the aim of this study was to identify the dominant bacterial pathogen common to external ocular infections, and to assess the in vitro drug susceptibility patterns of these isolates to commonly prescribed antibiotics among patients with external ocular infections.

## Methods

### Study setting and population

A cross-sectional study was conducted from February to May 2014 at Borumeda hospital which is located in Dessie town, Northeast Ethiopia. All patients who had sign and symptoms of external ocular infections were included as study populations. One hundred and sixty consecutive patients attending the Borumeda hospital from February to May 2014 were included in this study. Patients who had treatment with antibiotics within the last 5 days or who had undergone previous ocular surgery within the last 7 days of recruitment of study subjects were excluded from the study.

### Data collection

#### Soci-odemographic and clinical characteristics

Socio-demographic and clinical data were collected by trained optometrist from each study participants using pretested structured questionnaire. To identify the clinical picture of external ocular infections all patients were examined using a slit-lamp bio-microscope and diagnosed by ophthalmologist.

### Sample collection and Laboratory Investigation

#### Sample collection

After detailed ocular examinations, external ocular sample were collected by swabbing the purulent conjunctivitis. Briefly, patient was requested to look up, lower eye lid was pulled down and then samples were collected. The sample collector holds the palpebra apart and gently collects discharge from the surface of the eye using sterile cotton swab that has been pre-moistened with sterile saline. The sterile normal saline moistened swab was rubbed over the lower conjunctival sac from medial to lateral side and back again. Purulent material in cases of dacryocystitis was collected by everted puncta then applying pressure over the lacrimal sac area from the infected eye [16, 17]. The swab was immersed in 3 ml of brain heart infusion (BHI), placed in a cold box and transported to Dessie Regional Laboratory for investigation.

### Isolation and Identification of bacterial pathogens

Specimens were inoculated on to MacConkey agar, Mannitol Salt Agar, Blood agar and Chocolate agar (Oxoid Ltd Basingstoke, Hampshire, UK) plates and incubated at 37 °C for 48 hours. All plates were initially examined for growth after 24 hours and cultures with no growth were incubated for further 48 hours. After getting pure colonies, further identification were conducted using standard microbiological techniques, which include Gram stain, colony morphology and biochemical tests. Presumptive Gram negative bacteria were identified using triple sugar iron agar, citrate utilization test, lysine decarboxylase test, urease test and indole test and Gram positive bacteria were identified using catalase, coagulase, bacitracin and optochin tests [18].

### Antimicrobial susceptibility test

Antimicrobial susceptibility test was carried out on each identified bacterium using disc diffusion method on Muller Hinton agar (Oxoid Ltd Basingstoke, Hampshire, UK) based on clinical and laboratory standard institute (CLSI) 2014 guideline [19]. Briefly, 3–5 colonies of the test organism was emulsified in 5 ml of nutrient broth and mixed gently. The suspension was incubated at 37 °C until the turbidity of the suspension becomes adjustedto 0.5 McFarland standards. The suspension was uniformly rapped on to Mueller-Hinton agar for non-fastidious organisms and Mueller-Hinton agar with defibrinated sterile sheep blood (10 % V/V) for fastidious organisms. The antimicrobial impregnated disks were placed using sterile forceps on the agar surface and the plates were incubated at 37 °C for 24 hours and the zone of inhibition was determined.

### Quality control

Prior to actual data collection comprehensiveness, reliability and validity of questionnaires were pre-tested on ten patients at Dessie Referral Hospital eye clinic. All specimens were collected following standard operating procedure for ophthalmic specimen collection. The sterility of culture media was ensured by incubating 5 % of each batch of the prepared media at 37 °C for 24 hours. Performances of all prepared media were also checked by inoculating standard-strains such as *Escherichia coli* (ATCC 25922), *Staphylococcus aureus* (ATCC 25923) and *Pseudomonas aeruginosa* (ATCC 27853) obtained from Ethiopian Public Health Institute, Addis Ababa, Ethiopia [20]. The qualities of biochemical testing procedures were checked by these reference strains.

### Data analysis

Data was checked for completeness, coded, and first entered in to EPI-info version 7, then it was rechecked and transferred to Statistical Package for Social Science (SPSS) version 20 for analysis. The occurrence of bacterial isolates from the ophthalmic samples and their antimicrobial susceptibility pattern were used as dependant variables where as age, sex, occupation, educational status, and residence were used as independent variables during data analysis. Bivariate and multivariate logistic regression analyses were used to assess the possible risk factors bacterial external ocular infections. *P*-value < 0.05 at 95 % CI was considered statistically significant.

### Ethical consideration

Ethical approval was obtained from ethical review committee of School of Biomedical and Laboratory Sciences, College of Medicine and Health Sciences, University of Gondar, prior to data collection. Permission was taken from Borumeda Hospital administrators. Written informed consent was obtained from each individual after the purpose of the study explained. For children, consent was obtained from the guardian of the child who came to the hospital together with the child. Participants were told that they had full right to participate or not and they were also informed that all the data obtained from them would be kept confidential using codes instead of any personal identifiers. Any study participants who were positive for bacterial pathogens were referred to the ophthalmology clinicians for treatment.

## Results

### Socio-demographic characteristics of the study participants

A total of 160 patients were enrolled in this study. The majority of the study subjects were males (94/160; 58.8 %). The mean age of the study participants was 55.11 (SD ± 17.85) years. Most of the study participants were illiterate (132, 84.5 %), majority (75 %) were rural dwellers and 63.3 % (106/160) were farmer by their occupation (Table 1).

**Table 1:** Socio-demographic Characteristics of the study participants

Characteristics	Frequency	Percentage
Age in years		
≤ 18	8	5
19–45	36	22.5
46–65	45	28.1
> 66	71	44.4
Residence		
Rural	120	75.0
Urban	40	25.0
Sex		
Male	94	58.8
Female	66	41.3
Occupation		
Employed	14	8.8
Farmer	106	66.3
Merchant	11	6.9
Hose wife	14	8.8
Other	15	9.4
Educational status		
Unable to write and read	132	82.5
Write and read only	26	16.3
Primary school & above	2	1.3

### Clinical findings

**Fig. 1:**
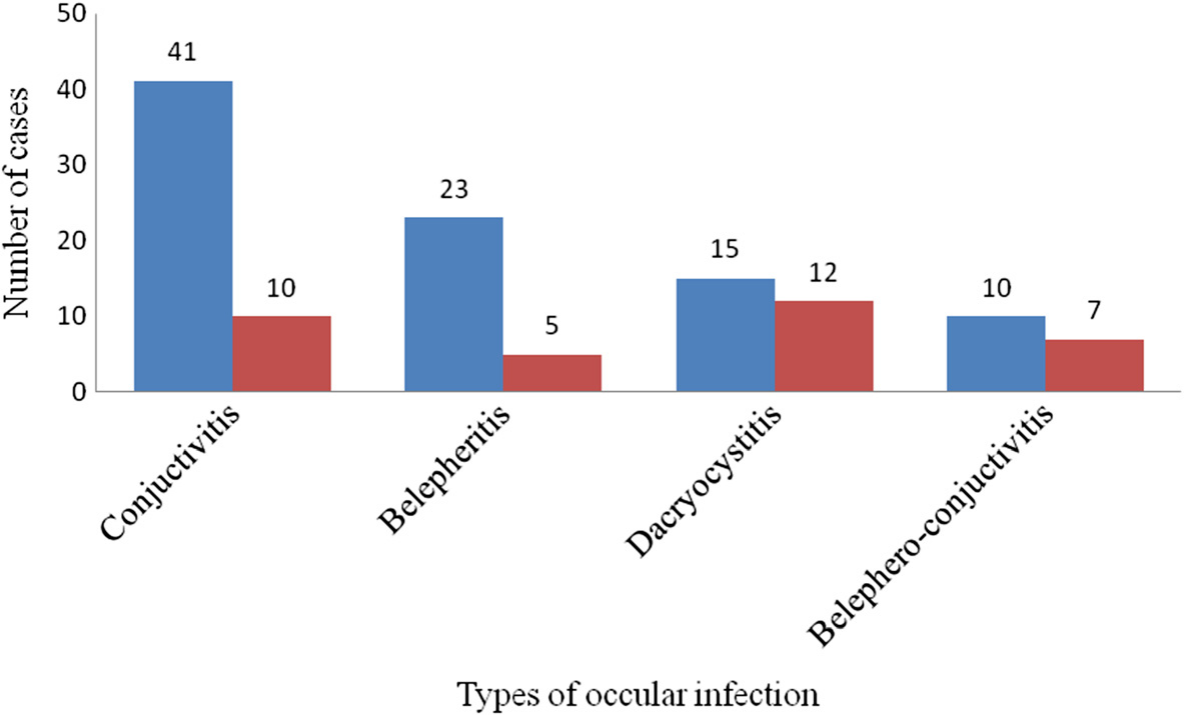
Prevalence of the different types of external eye infection by sex

In this study, 43.1 % (69/160) patients were suffering from conjunctivitis followed by blepharitis (29.4 %, 47/160). The dominant type of ocular infection among male patients was conjunctivitis (41/94; 43.6 %) were as in female patients a higher cases of dacryocystitis (12.3 %; 7/66) was observed. When the different types of eye infection were stratified by sex, higher prevalence of blepharitis and conjunctivitis cases were observed among male patients than females but the prevalence of dacryocystitis was higher among female patients (Fig. 1).

### Prevalence of bacterial pathogens and associated risk factors

Among 160 ocular specimens subjected to culture, 95 (59.4 %) were positive for different bacterial species. Among the culture positive isolates, 93.7 % (89/95) were Gram positive and 6.3 % (6/95) Gram negative bacteria. Coagulase negative *Staphylococci* (CoNS) was the predominant pathogen (57.3 %; *n* = 51/89) followed by *Staphylococcus aureus* (23.6 %, *n* = 21/89)) and *Streptococcus pneumoniae* (11.2 %, *n* = 10/89). Among patients clinically categorized as blepharitis (*n* = 47) and conjunctivitis (*n* = 69), CoNS were the most common isolates (46.8 %; *n* = 22/47 and 30.4 %; *n* = 21/69 respectively). Nine out of the ten *Streptococcus pneumoniae* isolates were found among patients clinically categorized as conjunctivitis and Belpharo- conjunctivitis. However, a comparatively higher prevalence of bacterial pathogens was found among patients suffering from dacryocystitis (80 %; 8/10) (Table 2).

**Table 2:** Prevalence of bacterial pathogens across the different clinical features of ocular infections

	Type of eye infection				Total (%)
Bacterial isolate	Belpharitis	Conjuctivitis	Belpharo-conjunctivitis	Dacrocystitis	
	(*n* = 47)	(*n* = 69)	(*n* = 34)	(*n* = 10)	
*CoNS*	22(46.8 %)	21(30.4 %)	7(20.6 %)	1(10 %)	51(31.9 %)
*S. aureus*	6(12.8 %)	6(8.7 %)	7(20.6 %)	2(20 %)	21(13.1 %)
*S. pneumoniae*	1(2.1 %)	7(10.1 %)	2(5.9 %)	0	10(6.2 %)
*Viridian streptococci*	0	2(2.9 %)	1(2.9 %)	0	3(1.9 %)
*S. pyogenes*	0	3(4.3 %)	1(2.9 %)	2(20 %)	6(3.8 %)
*S. agalactiae*	0	0	1(2.9 %)	0	1(.6 %)
*Enterococci spp*	0	0	1(2.9 %)	0	1(.6 %)
*E.coli*	0	0	0	1(10 %)	1(.6 %)
*p. aeruginosa*	0	1(1.4 %)	0	0	1(0.6 %)
*K. pneumoniae*	0	0	1(2.9 %)	0	1(.6 %)
*Proteus m.*	0	1(1.4 %)	0	1(10 %)	2(1.2 %)
*Salmonella spp.*	0	0	0	1(10 %)	1(.6 %)
*Enterobacter spp.*	1(2.1 %)	0	0	0	1(.6 %)
Total	30(63.8 %)	38(55.1 %)	19(55.9 %)	8(80 %)	95(100 %)

In this study, occupation, residence, education, frequency of face washing, the occurrence of systemic disease, and cigarette smoking were used as possible risk and predisposing factors for ocular infection. However, bivariate logistic regression analysis showed no statistically significant association of the occurrence of bacterial infection with any of the expected risk factors (Table 3).

**Table 3:** Bivariate analysis of possible risk factors and their association with the prevalence of bacterial infection of the eye among ophthalmic patients

Variables		Bacterial isolate		COR (95 % CI)	*P*-value
		Yes	No		
Male		57	37	1	
Female		38	28	1.24(0.66–2.59)	0.57
Age	<45	20	24	1	
	45–55	17	13	1.39(0.46–4.2)	0.56
	>55	58	28	2.3(0.88–6.04)	0.09
Residence					
Urban		20	20	1	
Rural		75	45	1.8(0.69–4.7)	0.23
Educational status					
Can’t read and write		80	52	0.46(0.02–10.67)	0.63
Read and write		14	12	0.83(0.04–17)	0.9
Primary school and above		1	1	1	
Occupation					
Government employee		10	4	1	
Farmer		65	41	1.8(0.42–7.8)	0.043
Others		20	20	0.95(0.37–2.45)	0.92
Frequency of face washing					
More Frequently		17	14	1	
Frequently		16	23	0.54(0.19–1.55)	0.25
Less frequently		62	23	1.53(0.63–3.)	0.35
Smoking habit					
Yes		9	1	6.34(0.72–56.14)	0.097
No		86	64	1	
Presence any underlying disease					
Yes		11	10	0.89(0.33–2.4)	0.81
No		84	55	1	

### Antimicrobial susceptibility patterns of bacterial isolates

The antimicrobial susceptibility patterns of bacterial isolates from ophthalmic patients showed that a significant number of bacterial isolates were resistant to one or more than one antimicrobials but all the Gram positive bacteria were sensitive to vancomycin. The drug susceptibility pattern of the Gram positive bacteria (*n* = 89) showed that 93.3 % (83/89) and 100 % of the isolates were sensitive to cefoxitin and vancomycin respectively. However, more than half of the Gram positive isolates (48/89; 53.9 %) and the other 34.7 % (38/91) showed resistance against amoxicillin and ampicillin respectively. Cefoxitin resistance was observed among 4 isolates (7.8 %) of CoNS. There was no drug resistant strain among the *Viridian streptococci* and *Streptococcus agalactiae* isolates (Table 4)*.* The drug susceptibility patterns of the Gram negative bacterial isolates showed that 5 out of 6 (83.3 %) were susceptible to gentamicin. However, majority of Gram negative bacteria isolates (4/6; 66.7 %) were resistance to tetracycline, norfloxacin, ceftriaxone, and ciprofloxacin (Table 5).

**Table 4:** Antimicrobial sensitivity pattern of Gram positive bacteria isolated from ophthalmic patients

Organisms isolated (*n* = 89)		Antibiotics tested								
		VAN	PE	DO	AMP	AML	DA	FOX	CIP	CRO
*S. pneumoniae*	S	10(100 %)	6(60 %)	8(80 %)	7(70 %)	4(40 %)	9(90 %)	9(90 %)	8(80 %)	10(100 %)
	I	0	0	0	0	0	0	0	0	0
	R	0	4(40 %)	2(20 %)	3(30 %)	6(60 %)	1(10 %)	1(10 %)	2(20 %)	0
CoNS	S	51(100 %)	37(72.5 %)	37(72.5 %)	37(72.5 %)	26(51 %)	47(92.2 %)	47(92.2 %)	47(92.2 %)	45(88.3 %)
	I		0	1(2 %)	0	5(9.8 %)	0	0		
	R		14(27.5 %)	13(25.5 %)	14(27.5 %)	20(39.2 %)	4(7.8 %)	4(7.8 %)	4(7.8 %)	6(11.8 %)
*S.aureus*	S	21(100 %)	14(66.7 %)	12(57.1 %)	11(52.4 %)	7(33.3 %)	17(81 %)	20(95.2 %)	19(90.5 %)	20(95.2 %)
	I	0	0	2(9.5 %)	0	1(4.8 %)	1(.6 %)	0		
	R	0	7(33.3 %)	7(33.3 %)	10(47.6 %)	13(61.9 %)	3(14.3 %)	1(4.8 %)	2(9.5 %)	1(4.8 %)
*S. pyogenes*	S	6(100 %)	4(66.7 %)	5(83.3 %)	2(33.3 %)	2(33.3 %)	5(83.3 %)	6(100 %)	6(100 %)	5(83.3 %)
	I	0	0	0	0	0	1(16.7 %)	0	0	0
	R	0	2(33.3 %)	1(16.7 %)	4(66.7 %)	4(66.7 %)	0	0	0	1(16.7 %)
*Enterococci spp.*	S	1(100 %)	1(100 %)	1(100 %)	1(100 %)	1(100 %)	1(100 %)	1(100 %)	1(100 %)	1(100 %)
	I	0	0	0	0	0	0	0	0	0
	R	0	0	0	0	0	0	0	0	0

CoNS* Coagulase Negative Staphylococcus, *S* sensitive, *I* Intermediate, *R* Resistance, *Amp-* Ampicillin, *CRO-* Ceftriaxone, *FOX-* Cefoxitin, *CIP-* Ciprofloxacin, *DA-* Clindamycin, *AMP-* ampicillin, *AML-* amoxicillin, *PE-* penicillin, *VAN-* Vancomycin

**Table 5:** Antimicrobial Sensitivity Pattern of Gram Negative bacteria isolated from ophthalmic patients

Organism(*n* = 6)	Sensitivity	Antibiotic tested				
		CRO	NOR	TE	GS	CIP
*P. aeruginosa*	S	1	1	1	1	1
	I	0	0	0	0	0
	R	0	0	0	0	0
*P. mirabilis*	S	2	2	1	2	2
	I	0	0	0	0	0
	R	0	0	1	0	0
*E. coli*	S	1	0	0	0	0
	I	0	0	0	0	0
	R	0	1	1	1	1
*Salmonella spp.*	S	1	1	1	1	1
	I	0	0	0	0	0
	R	0	0	0	0	0
*Enterobacter spp.*	S	0	0	1	1	0
	I	0	0	0	0	0
	R	1	1	0	0	1
*K. pneumoniae*	S	0	0	0	1	0
	I	0	0	0	0	0
	R	1	1	1	0	1

*CIP-* ciprofloxacin, *CRO-* ceftriaxone, *NOR*- norfloxacin, *CIP-* Ciprofloxacin, *GS-* Gentamycin, *TE-* tetracycline, *S* sensitive, *I* Intermediate, *R* Resistance

## Discussion

The organisms that cause ocular infection are generally exogenous. However, in certain circumstances they gain accesses to enter the eye and cause infection. In this study, the prevalence of bacteria caused eye infection was 59.4 % and the result is very comparable with other previous study reports conducted in Gondar and India (60.8 % and 58.8 % respectively) [21, 14]. However, higher prevalence of bacteria caused ophthalmic infection (74.7 %) was reported in Jimma, South western parts of Ethiopia [2] and comparatively lower prevalence (47 %) in Addis Ababa, Ethiopia [22]. In this study, the majority of patients were rural dwellers and farmer by occupation. The risk of agricultural predominance and vegetative corneal injury in bacterial keratitis increase susceptibility to corneal infection [13]. Moreover, co-existing ocular disease predisposing bacterial keratitis was frequently recorded among non-agricultural workers (84.3 %) compared with agricultural workers (45.2 %). However, history of corneal injury predisposing to fungal keratitis was frequently recorded among agricultural workers than non-agricultural workers [23]. Although frequency of face washing was not associated with bacteria caused external ocular infection in the present study, Ejere *et al.* [24] found a statistically significant effect of face washing combined with topical tetracycline application in reducing severe active trachoma compared to topical tetracycline application alone.

In the current study, conjunctivitis was the dominant type of eye infection (43.1 %) followed by blepharitis (29.4 %). Conjunctivitis was also found the primary ocular morbidity accounting for 29 %, followed by cataract (16.3 %), presbyopia (15.4 %), refractive errors (7.9 %), and blepharitis (7.5 %) during an ophthalmic outreach campaign in Kersa town, Southeastern Addis Ababa, Ethiopia [25]. The prevalence of conjunctivitis was also found higher among male patients where as a higher cases of dacryocystitis observed among female patients in the current study. Higher rates of both acute and chronic dacryocystitis have been reported in previous studies among women [26, 27]. Data of the current study showed that external ocular infections were predominantly seen among male patients. This might be due to their outdoor activities and that was also supported by similar reports from Jimma and Gondar, Ethiopia [2, 22].

Gram positive bacteria were the dominant isolate (55.6 %) in the current study. This is also supported by other studies conducted in Ethiopia [22] and Nigeria [28] suggestive of Gram positive cocci as a primary cause of external ocular infection. Among the Gram positive bacteria, CoNS was the most predominant pathogen with an overall prevalence of 31.9 % (*n* = 51/160). Previous reports from India [29], Uganda [30] and Ethiopia [22] showed that CoNS was the most predominant isolated pathogen from ocular infections. Other study reports showed that *Staphylococcus aureus* as predominant isolate [31]. *Streptococcus pneumoniae* was also reported as commonest Gram positive bacterial pathogen in external ocular infections from Malaysia and Ethiopia [31, 32]. The occurrence of different bacteria as an etiological agent for external ocular infection signifies differences in the environmental conditions, the standard of personal hygiene, age and site of infection.

The prevalence of Gram negative bacteria as etiological agents of ophthalmic disease in the current study can be graded lower as only 3.8 % (*n* = 6/160) of the patients were positive for these bacterial pathogens. Previous to this report, the prevalence of Gram negative bacterial pathogens among patients suffering from ophthalmic disease in Jimma was reported 48 % [7]. The low prevalence of Gram negative enteric bacteria in the present study could be due to effective personal hygiene as the most important mode of transmission for enteric pathogens is fecal-oral contamination of the eye. During data collection, we noticed that health extension workers deliver health education about latrine usage and west disposal in the study area. Moreover, there are reports that documented the main cause for Gram negative bacteria caused ocular infection is contact lens wearing [33]. In the present study, none of the patients had contact lens wearing history.

The drug susceptibility patterns of Gram positive cocci bacterial isolates showed a 100 % sensitivity pattern to vancomycin. However, most of the isolates were resistant to ampicillin and amoxicillin and this was very similar to the previous report from Gondar [22]. Reduced efficacy of ampicillin and amoxicillin could possibly be due to the frequent usage of these drugs by patients as these antibiotics are commonly used by many patients with and without prescription.

Most of Gram negative isolates were sensitive to gentamicin (*n* = 5; 83.3 %) but resistant to tetracycline, norfloxacylin, ceftriaxone, and ciprofloxacin (*n* = 4; 66.7 %). Several reports also showed similar patterns of drug resistance among Gram negative bacteria [34]. *Pseudomonas* species was among the highly drug resistant isolates reported previously and found very challenging organism to treat [35]. However, the single *Pseudomonas aeruginosa* isolate of the current study was sensitive to all antibiotics. Frequent isolation of drug resistant bacteria might be due to an irrational use of antimicrobial agents. In Ethiopia, it is a common practice that antimicrobials can be purchased without prescription, which leads to misuse of antibiotics. This may contribute to the emergence and spread of antimicrobial resistance [2, 10]. Other factors may include availability of the suboptimal quality or substandard antimicrobial drugs, increased usage of a particular antimicrobial agent, poor sanitation, contaminated food and cross-contamination from humans or animals [36, 37]. Other contributing factors may include improper dosage regimen during administration which includes difficulty of administration of drops of antibiotics in day time use for adult populations and children [38]. The limitation of this study was that due to resource constraints anaerobic bacteria and *Chlamydia trachomatis* caused ocular infections were not investigated.

## Conclusion

The prevalence of bacterial infection among patients with external ocular infection in Borumeda hospital, Northeast Ethiopia was high (59.4 %). The predominant isolates were coagulase negative *Staphylococci.* High antibiotic resistance to commonly prescribed antibiotics was observed. Exceptionally high amoxicillin resistant Gram positive bacteria were identified. Therefore, to prevent the increasing rate of antimicrobial resistance identification of bacteria through culture methods and conducting drug susceptibility test should be practiced as a routine diagnostic procedure.

## References

[1] Ramesh S, Ramakrishnan R, Bharathi M, Amuthan M, Viswanathan S (2010). Prevalence of bacterial pathogens causing ocular infection in south India. Indian J Pathol Microbiol.

[2] Tesfaye T, Beyene G, Gelaw Y, Bekele S, Saravanan M (2013). Bacterial Profile and Antimicrobial Susceptibility Pattern of External Ocular Infections in Jimma University Specialized Hospital, Southwest Ethiopia. Am J Ophthalmol..

[3] Sharma S (2011). Antibiotics and Resistance in Ocular Infections. Indian J Med Microbiol..

[4] Sthapit PR, Tuladhar NR, Marasini S, Khoju U, Thapa G (2011). Bacterial Conjunctivitis and Use of Antibiotics in Dhulikhel Hospital - Kathmandu University Hospital. Kathmandu Univ Med J..

[5] Whitcher JP, Srinivasan M, Upadhyay MP (2001). Corneal blindness: a global perspective. Bull World Health Organ..

[6] Marilyn H and Charles S. Conjunctivitis: Bacterial, Viral, Allergic and Other Types. All about vision.com. Access Media group LLC, March 2014.

[7] Sinha R, Sharma N, Vajpayee RB. Corneal Blindness -- Present Status. Cataract & Refractive Surgery Today. 2005;59–61.

[8] Lemp MA, Nichols KK (2009). Blepharitis in the United States 2009: a survey-based perspective on prevalence and treatment. TOS.

[9] Azari AA, Barney NP (2013). Conjunctivitis A Systematic Review of Diagnosis and Treatment. JAMA..

[10] Chaudhry IA, Shamsi FA, Al-Rashed W (2005). Bacteriology of chronic dacryocystitis in a tertiary eye care center. Ophthal Plast Reconstr Surg..

[11] Mariotti SP, Pascolini D, Rose-Nussbaumer J (2009). Trachoma: global magnitude of a preventable cause of blindness. Br J Ophthalmol..

[12] Gopinathan U, Sharma S, Garg P, Rao G (2009). Review of epidemiological features, microbiological diagnosis and treatment outcome of microbial keratitis: Experience of over a decade. Indian J Ophthalmol.

[13] Bharathi MJ, Ramakrishnan R, Meenakshi R, Padmavathy S, Shivakumar C, Srinivasan M (2007). Microbial keratitis in South India: influence of risk factors, climate, and geographical variation. Ophthal Epidemiol..

[14] Birhane Y, Worku A, Bejiga A, Adamu L, Alemayehu W, Bedri A (2007). Prevalence and causes of blindness and low vision in Ethiopia. Ethiop J Health Dev.

[15] Brown L (2007). Resistance to ocular antibiotics: an overview. Clin Exp Optom..

[16] Baron EJ, Miller JM, Weinstein MP, Richter SS, Gilligan PH, Thomson RB (2013). A guide to utilization of the microbiology laboratory for diagnosis of infectious diseases: 2013 recommendations by the Infectious Diseases Society of America (IDSA) and the American Society for Microbiology (ASM). Clin Infect Dis..

[17] Sharma S (2012). Diagnosis of infectious diseases of the eye. Eye.

[18] Cheesbrough M (2006). District Laboratory Practice in Tropical Countries Part I.

[19] Clinical and Laboratory Standard Institute (CLSI). Performance standards for antimicrobial susceptability testing; twenty-forth informational supplement CLSI document M100-S24. CLSI 2014.

[20] Vandepitte J, Verhaegen J, Engbaek K, Rohner P, Piot P, Heuck CC (2003). Basic Laboratory Procedures in Clinical Bacteriology.

[21] Muluye D, Wondimeneh Y, Moges F, Nega T, Ferede G (2014). Types and drug susceptibility patterns of bacterial isolates from eye discharge samples at Gondar University Hospital, Northwest Ethiopia. BMC Research Notes.

[22] Nigatu N (2004). Pattern of Microbial agents of External Ocular Infections in Federal Police Hospital And Minilik II Memorial Hospital.

[23] Bharathi MJ, Ramakrishnan R, Meenakshi R, Shivakumar C, Lional RD (2009). Analysis of the risk of factors predisposing to fungus, bacterial and Acanthamoeba keratitis in South India. Indian J Med Res..

[24] Ejere H, Alhassan MB, Rabiu M. Face washing promotion for preventing active trachoma. Cochrane Database Syst Rev. 2004;(3):CD003659. http://www.ncbi.nlm.nih.gov/pubmed/15266493. 10.1002/14651858.CD003659.pub2 15266493

[25] Zelalem A (2013). A study of ocular morbidity of patients attending ophthalmic ophthalmic outreach services in rural Ethiopia. Int J Med Med Sci.

[26] Ali MJ, Joshi SD, Naik MN, Honavar SG (2015). Clinical profile and management outcome of acute dacryocystitis: two decades of experience in a tertiary eye care center. Semin Ophthalmol.

[27] Mills DM, Bodman MG, Meyer DR, Morton AD (2007). The microbiologic spectrum of dacryocystitis: a national study of acute versus chronic infection. Ophthal Plast Reconstr Surg.

[28] Iwalokun BA, Oluwadun A, Akinsinde KA, Niemogha MT, Nwaokorie FO (2011). Bacteriologic and plasmid analysis of etiologic agents of conjunctivitis in Lagos, Nigeria. J Ophthalmic Inflamm Infect..

[29] Summaiya M, Neeta K, Sangita R (2012). Ocular Infections: Rational Approach to Antibiotic Therapy. National J Med Res.

[30] Mshangila B, Paddy M, Kajumbula H, Ateenyi-Agaba C, Kahwa B, Seni J (2013). External ocular surface bacterial isolates and their antimicrobial susceptibility patterns among pre-operative cataract patients at Mulago National Hospital in Kampala, Uganda. BMC Ophthalmol.

[31] Ah Chaudhry IA, Shamsi FA, Al-Rashed W (2005). Bacteriology of chronic dacryocystitis in a tertiary eye care center. Ophthal Plast Reconstr Surg..

[32] Kebede A, Adamu Y, Bejiga A (2010). Bacteriological study of dacryocystitis among patients attending in Menelik II Hospital, Addis Ababa, Ethiopia. Ethiop Med J.

[33] Epling J (2012). Bacterial conjunctivitis. Clin Evid.

[34] Biradar S, Chandrashekhar D, Gangane R, Chandrakanth C, Biradar K, VinodKumar C (2012). Spectrum of microbial keratitis and antimicrobial susceptibility at tertiary care teaching hospital in North Karnataka. Int J Pharm Biomed Res.

[35] Willcox M (2011). Review of resistance of ocular isolates of Pseudomonas aeruginosa and staphylococci from keratitis to ciprofloxacin, gentamicin and cephalosporins. Clin Exp Optom.

[36] Ahanna UU (2009). Common Bacterial Isolates from Infected Eyes. JNOA..

[37] Joseph S, Bertino J (2009). Impact of Antibiotic Resistance in the Management of Ocular Infections: The Role of Current and Future Antibiotics. Clin Ophthalmol.

[38] Kimberly K. How to manage bacterial eye infections. Rev Optometry 2011, 84–91. http://www.reviewofoptometry.com/continuing_education/tabviewtest/lessonid/107608/.

